# Brazilian red propolis: phytochemical screening, antioxidant activity and effect against cancer cells

**DOI:** 10.1186/s12906-015-0888-9

**Published:** 2015-10-14

**Authors:** Izabel Cristina Gomes de Mendonça, Isabel Cristina Celerino de Moraes Porto, Ticiano Gomes do Nascimento, Naiana Soares de Souza, José Marcos dos Santos Oliveira, Rodolfo Elleson dos Santos Arruda, Kristiana Cerqueira Mousinho, Aldenir Feitosa dos Santos, Irinaldo Diniz Basílio-Júnior, Abhishek Parolia, Francisco Stefânio Barreto

**Affiliations:** School of Dentistry, Cesmac University Center, Rua Cônego Machado, 918, Farol, CEP: 57051–160, Maceió, Alagoas Brazil; School of Nursing and Pharmacy, Federal University of Alagoas, Campus A. C. Simões, University City, Maceió, 57072-970 AL Brazil; School of Dentistry, International Medical University, Bukit Jalil , 57000 Kuala Lumpur, Malaysia; Laboratory of Experimental Oncology (LOE), Medical School, Federal University of Ceará, Rua Cel. Nunes de Melo 1127, 60430-270 Fortaleza, CE Brazil

**Keywords:** Red propolis, Phenolic compounds, Antioxidant activity, Cytotoxic activity

## Abstract

**Background:**

The implementation of new public healthcare models that stimulate the use of natural products from traditional medicine, as a so-called integrated medicine, refers to an approach that use best of both conventional medicine and traditional medicine. Propolis is a widely used natural product by different ancient cultures and known to exhibit biological activities beneficial for health. The large number of studies conducted with propolis had shown that its chemical composition differs as a function of the climate, plant diversity and bee species and plays an important role on its therapeutic properties. The aim of this study was to analyse the phytochemical profile of the ethanolic extract of red propolis (EEP) and its fractionation, antioxidant action of EEP and its fractions hexane, cloroform and ethyl acetate and cytotoxic activity of EEP on human tumour cell lines SF-295 (glioblastoma), OVCAR-8 (ovary) and HCT-116 (colon).

**Methods:**

EEP was obtained by maceration with absolute ethanol, then it was concentrated in rotaevaporator up to complete evaporation of the solvent. The crude extract was fractionated with hexane, ethyl acetate, chloroform and methanol and they were subjected to phytochemical screening and total phenolic compounds. Antioxidant activity of EEP and fractions was done by means of the 2,2-diphenyl-1-picryhydrazyl (DPPH) method. Biomarkers of red propolis were identified by LC-Orbitrap-FTMS. To assess cytotoxic activity of the extract, cells were exposed to EEP over 72 h. Cell viability was assessed by means of MTT assay. The percentage of cell growth inhibition (IC_50_) was analysed by means of non-linear regression, and the absorbance values of the various investigated concentrations were subjected to one-factor analysis of variance (ANOVA) followed by Tukey’s or Tamhane’s tests (α = 0.05).

**Results:**

The results obtained using phytochemical screening and LC-Orbitrap-FTMS indicated the presence of phlobaphene tannins, catechins, chalcones, aurones, flavonones, flavonols, xanthones, pentacyclic triterpenoids and guttiferones in Brazilian red propolis. EEP and its hexane, chloroform and ethyl acetate fractions obtained by liquid-liquid partitioning exhibited satisfactory antioxidant percentages. EEP (IC_50_ < 34.27 μg/mL) exhibited high levels of cytotoxicity on all human tumour cell lines tested when compared to negative control.

**Conclusions:**

C-Orbitrap-FTMS was useful to establish the chemical profile of the red propolis. Brazilian red propolis has antioxidant properties and decreases substantially the percentage of cell survival of human tumour cells; thus, it has potential to serve as an anticancer drug.

## Background

The use of medicinal plants, phytotherapeutics and organotherapy products in the treatment of diseases is growing worldwide and thus represents a promising market for the development of new drugs and the treatment of complex diseases, such as cancer [1–5].

Propolis has been known since centuries for its healing properties but recently it has captured the attention of scientists due to its wide spectrum activities that can be used in the complementary and alternative medicine [6–8]. Consistently, the number of studies conducted with this substance has exhibited a significant increase in countries without the traditional use of natural medicine [2, 9–12].

Propolis is a resinous substance produced from plant buds by bees from the species *Apis mellifera* and serves as a natural barrier to protect hives from invading parasites, bacteria and viruses [13]. Although the composition of propolis varies as a function of its botanical and geographical origin [8, 9], it usually includes beeswaxes, balsams, vitamins, minerals, essential oils and resin, which are rich in secondary plant metabolites such as phenolic compounds [8, 13, 14]. Propolis is considered an organotherapy product because it contains organic secretions of the bees that produce it [15]. The most widely known pharmacologically active chemical components in propolis are flavonoids, isoflavonoids, phenolic acid, terpens, xanthones, propolones and guttiferones [8, 13, 14, 16], which account for its antimicrobial [10, 17, 18], anti-inflammatory [10, 17, 19], antioxidant [19], antiviral [19], antifungal [20, 21] and anticancer actions [2, 3, 11, 22], among other.

Climate variations might induce changes in the concentration of bioactive compounds of plants, with consequent alterations in the biological activity of the various types of propolis [23, 24]. Although, therapeutic standardisation of propolis is challenging, and the relationship between definite types of propolis and specific biological activities is difficult to establish [14], the presence of a significant amount of one specific compound might lead to the expectance that the extract has the potential to show bioactivities linked to this potential [7, 8]. The chemical composition of Brazilian propolis is quite different from that of propolis from European countries as a function of the tropical climate, plant diversity and bee species, the latter resulting from the crossing of European and African species. Those factors play a relevant role in the physical, chemical and biological properties of propolis [25].

Park et al. [26] have identified and classified 13 different types of propolis in Brazil based on their physicochemical characteristics. Red propolis is a type of Brazilian propolis found in beehives close to mangrove swamps in the northeast states of Alagoas, Sergipe, Bahia, Pernambuco and Paraíba [26, 27]. The botanical origin of this newly found red-hued propolis is a leguminous plant known as *Dalbergia ecastophyllum*, which grows abundantly in the mangrove swamps of Alagoas State. Recent studies that sought to characterise the Brazilian red propolis found molecules, such as elemicin, isoelemicin, methyl isoeugenol, methyl eugenol, formononetin, biochanin A, isoliquiritigenin, liquiritigenin, medicarpin, homopterocarpan, quercetin and vestitol, that allow it to be distinguished from other types of Brazilian propolis [19, 27, 28].

Red propolis exhibits more intense cytotoxic action and inhibition of human leukaemia cells growth compared with green propolis [2]. The cytotoxic activity of red propolis on six different tumour cell lines was similar to that of anticancer drugs, such as 5-fluorouracil and doxorubicin [3]. Costa e Silva [29] has verified the action of Brazilian red propolis on murine melanoma (BB16F10), multiple human melanoma (RPMI 8226), promyelocytic leukemia (HL-60), chronic myeloid leukemia (K562) and human normal lung fibroblasts (MRC-5) cell lines. The results showed cytotoxic effect, ie, the propolis was able to inhibit the growth of all tumour cells by necrosis, with in average IC50 around 31.3 ug / mL in 24 h of exposure, whereas the IC50 after 24 h exposure of normal fibroblast cells to Brazilian red propolis was 36.4 μg / mL. Frozza et al. [30] evaluated cytotoxic activity of Brazilian red propolis on human laryngeal epidermoid cells carcinoma (Hep-2), human cervical adenocarcinoma cells (HeLa) and human normal epithelial embryonic kidney cells (Hek-293). Survival analysis for non-tumor cell line showed greater IC50 (>150 μg/mL) compared to tumor cell lines (Hep-2 – 63.48 μg/mL and HeLa – 81.40 μg/mL), suggesting an increased sensitivity that may correlate with the higher proliferative index of the tumor vs. normal cells. Studies of cytotoxicity *in vitro* are part of the initial screening to identify whether the tested substance interferes with both cell metabolism or cell survival. At this point it is too early to attest its safety. Although many studies prove the therapeutic and beneficial properties of propolis, toxicity studies *in vivo* and *in vitro* should be done to better support the safe use of propolis.

Therefore, the confirmed action of red propolis on tumour cells [2, 3, 22], together with the implementation of a new public healthcare model that stimulates the population’s use and interest in natural products point to the need to gather more information on this substance. The aim of this study was to analyse the phytochemical profile of the ethanolic extract of red propolis (EEP) and its fractionation, antioxidant action of EEP and its fractionation and cytotoxic activity of EEP on human tumour cell lines SF-295 (glioblastoma), OVCAR-8 (ovary) and HCT-116 (colon).

## Methods

### Propolis sample

Red propolis raw material (300 g) was collected from Marechal Deodoro city, State of Alagoas, Brazil. Propolis was collected from the Ilha do Porto apiary with geographical coordinates of South latitude: 9° 44.555′, West latitude: 35° 52.080′ and height of 18.1 m above sea level. The access and transportation of Brazilian red propolis was previously authorised by regulatory agencies for control of Brazilian Genetic Heritage and Biodiversity Conservation with protocol number of acceptance 010124/2012-8.

### Reagents

Roswell Park Memorial Institute 1640 (RPMI 1640) culture medium and foetal bovine serum (FBS) were purchased from Gibco Invitrogen (Karlsruhe, Germany). Penicillin/streptomycin, dimethyl sulphoxide (DMSO) and salt 3-(4,5-dimethyl-2-thiazole)-2,5-diphenyl-2-H-tetrazolium bromide (MTT) were purchased from Sigma Chemical (St. Louis, MO, USA). Folin-Ciocalteu reagent and 2,2-diphenyl-1-picryhydrazyl (DPPH) were purchased from Merck KGaA (Darmstadt, Germany). Methanol, hydrochloric acid, ethanol, ferric chloride, sodium hydroxide, chloroform, anhydrous sodium sulphate, sulphuric acid, sodium carbonate, gallic acid and ethyl acetate were purchased from Vetec Química Fina Ltda. (Duque de Caxias, RJ, Brazil).

### Cell culture

Tumour cell lines SF-295 (human glioblastoma), OVCAR-8 (ovary) and HCT-116 (colon) supplied by the National Cancer Institute (USA) were grown in RPMI 1640 medium supplemented with 10 % FBS, 2 mM glutamine, 100 μg/mL of streptomycin and 100 U/mL of penicillin in an incubator at 37 °C and 5 % CO_2_.

#### Preparation of experimental solutions

EEP was diluted in sterile, pure DMSO and then tested at a concentration of 50 μg/mL (reference solution) to establish its cytotoxic activity on SF-295, OVCAR-8 and HCT-116 cell lines.

To establish the half-maximal inhibitory concentration (IC_50_), the samples were tested in serial dilutions (0.09, 0.19, 0.39, 0.78, 1.56, 3.12, 6.25, 12.5, 25 and 50 μg/mL) of the reference solution in culture medium and 1 % DMSO using dilution factor 2. The wells for the negative control were filled with the solvent used to dilute EEP (1 % DMSO), and 0.5 μM doxorubicin, an anthracycline antibiotic widely used in the treatment of various types of cancer [31] was placed in the wells as a positive control.

#### Assessment of EEP cytotoxicity

The MTT assay was used to establish the cytotoxicity of EEP. SF-S95 and OVCAR-8 cells were plated at a concentration of 0.1 × 10^6^ cells/mL and the HCT-8 cells at a concentration of 0.7 × 10^5^/mL; the cells were then incubated with EEP in the above-mentioned dilutions for 72 h in an incubator at 37 °C with 5 % CO_2_. The cells were centrifuged, and the supernatant was removed. Next, 150 μL of MTT solution (methyl tetrazolium salt) was added to each well, and the plates were incubated under the above-mentioned conditions for three hours. Following dissolution of the precipitate with 150 μL of pure DMSO, the absorbance readings were performed using the plate reader ELISA Synergy (Bio Tek Instruments, Highland Park, Winooski, USA) at 595 nm. The test was performed in triplicate.

### Preparation of ethanolic extract of propolis (EEP)

Raw propolis (250 g) was manually grounded and placed in a flask with 600 mL of 80 % ethanol, which was placed on an agitator (Thornton, Model T14, USA) for 48 h. Then, the macerate (the liquid portion) was removed using a pipette, and the solid portion (wax) was discarded. The macerate was mixed with 600 mL of 80 % ethanol in a glass flask, placed on the agitator for 24 h. Then the resulting macerate was mixed again with 600 mL of 80 % ethanol and left for 24 h without agitation.

Next, the macerate was removed using a pipette, filtered through filter paper and subjected to distillation under reduced pressure in a rotary evaporator (Fisatom, São Paulo-Brasil) in a water bath at temperature 80–90 °C, pressure 650 mmHg and speed 80 rpm to remove the solvent. The EEP was then placed in a glass container and left for approximately three days for the residual solvent to evaporate; as a result, a solid mass (162 g) with viscous appearance was obtained.

### Liquid-liquid partitioning

EEP (10 g) was fractionated by means of liquid-liquid partitioning. For that purpose, the extract was dissolved in methanol and water in proportion 8:2 (v/v; volume in 50 mL). It was added 15 mL of water following of vigorous agitation and allowed to stand for a few minutes. The methanol/water phase was used as the basis for partitioning, using solvents: hexane (first), chloroform (second) and ethyl acetate (third) on this sequence according to the gradient of polarity and both with 100 mL. In this procedure a gentle agitation was used to avoid emergence of emulsions, resulting in the hexane, chloroform and ethyl acetate fractions, respectively. The volume of each fraction was subjected to distillation under reduced pressure in a rotary evaporator similar to the procedure of crude ethanolic extract of propolis (EEP). Solid masses were obtained for hexane fraction, chloroform fraction and ethyl acetate fractions after total evaporation of solvents. The EEP and its fractions were subjected to phytochemical prospection, assessment of the percentage of phenolic compounds and investigation of antioxidant activity. Cytotoxicity assay was used only for EEP.

### Phytochemical screening of red propolis extracts

#### Prospection of chemical components

Phytochemical screening was performed based on the methods suggested by Matos [32], which were adapted for the prospection of the following allelochemicals: phenols, pyrogallic tannins, phlobaphene tannins, anthocyanin, anthocyanidin, flavones, flavonols, xanthones, chalcones, aurones, flavononols, leucoanthocyanidins, catechins, flavanones, steroids, triterpenoids and saponins.

For phytochemical prospection, crude extract of EEP and its fractions were exactly weighted (100 mg) solubilized in absolute ethanol (40 mL) and performed the same procedure cited for phytochemical prospection. Then, aliquot of 35.0 mL of the samples (EPP and its fractions in separate test tubes) were divided in seven 3.0-mL parts, which were placed in numbered (1 to 7) and labelled test tubes, and one 10.0-mL part was placed in a beaker. The beaker was heated in water bath on a hot plate under agitation, until the liquid was fully evaporated, to assess steroids, triterpenoids and saponins.

#### Tests to assess phenols, pyrogallic tannins and phlobaphene tannins

In test tube #1, three drops of solution of ferric chloride (FeCl_3_) in alcohol were added to the EEP sample. Following agitation, the tube was inspected to detect changes in colour or abundant formation of dark-hued precipitate. Hues ranging from blue to red indicate the presence of phenol; bluish dark precipitate indicates the presence of pyrogallic (hydrolysable) tannins, and green precipitate indicates the presence of phlobaphene (condensed tannins or catechins) tannins. For the purpose of comparison, one blank test was performed with water and ferric chloride only.

#### Test to assess anthocyanin and anthocyanidin, flavones, flavonols and xanthones, chalcones and aurones and flavononols

Test tube #2 was acidulated with hydrochloric acid (HCl) to pH 3.0; test tube #3 was alkalinised to pH 8.5, and test tube #4 was alkalinised to pH 11 through the addition of sodium hydroxide (NaOH). The test tubes were inspected for changes in colour: a change to the colour red at pH 3, lilac at pH 8.5 and purplish-blue at pH 11 indicates the presence of anthocyanin and anthocyanidin; the colour yellow at pH 11 indicates the presence of flavones, flavonols and xanthones; red at pH 3 and purplish red at pH 11 indicates the presence of chalcones and aurones; and red-orange at pH 11 indicates the presence of flavononols.

#### Test to assess leucoanthocyanidins, catechins and flavanones

Test tube #5 was acidulated through addition of HCl to pH 2.0, and test tube #6 was alkalinised through addition of NaOH to pH 11.0. The tubes were heated using an alcohol lamp for 3 min. A change in colour to red at pH 2.0 indicates the presence of leucoanthocyanidins, to brown at pH 2.0 the presence of catechins, and to red-orange at pH 11 the presence of flavanones.

#### Tests to assess flavonols, flavanones, flavononols and xanthones

A small magnesium ribbon and 1.0 mL of concentrated HCl were added to test tube #7. The end of reaction was indicated by appearance of effervescence, following which, test tubes #5 and #7 (both acidulated) were compared, seeking to detect changes to or intensification of the red colour, which indicates the presence of flavonols, flavanones, flavononols and/or xanthones, either free or the corresponding glycosides.

#### Test to assess steroids and triterpenoids

The dry residue was extracted three times with 2.0 mL of chloroform and homogenised. The solution was filtered into a test tube, by dripping one drop at a time across a small funnel covered by cotton with a few decigrams of anhydrous sodium sulphate (Na_2_SO_4_). Next, 1.0 mL of acetic anhydride was added, and the tube was gently agitated before the addition of three drops of concentrated sulphuric acid (H_2_SO_4_). Following agitation, the test tube was inspected for changes in colour: evanescent blue followed by permanent green indicates the presence of free steroids; a hue ranging from brown to red indicates the presence of free pentacyclic triterpenoids.

#### Test to assess saponins

The chloroform-insoluble residue produced in the above-mentioned reaction was dissolved in 8.0 mL of distilled water, and the solution was filtered through cotton into a test tube. The test tube was then strongly agitated over three minutes and was inspected for formation of abundant persistent foam (head), which indicates presence of saponins (saponin glycosides).

### Assessment of total phenolic compounds

The EEP and its fractions were subjected to triplicate assessment at a concentration of 2.0 mg/mL. A total of 0.5 mL of 2 N Folin-Ciocalteu reagent and 1.0 mL of water were added per 0.5 mL of the propolis sample, and the tubes were agitated over two minutes. Next, 0.5 mL of 10 % sodium carbonate (Na_2_CO_3_) was added. Following incubation for two hours at room temperature with the tubes protected from light, absorbance was measured using a spectrophotometer (Model UV-1700, Shimadzu, Kyoto, Japan) at 750 nm. Methanol was used as blank [33].

Gallic acid (100 mg) was exactly weighted and transferred for volumetric flask (10 mL) and solubilised with methanol to obtain a stock solution (10.0 mg/mL). Gallic acid stock solution was diluted for concentrations of 1.0 mg/mL and solubilised with methanol to obtain work solution and aliquots of 0.150, 0.100, 0.050, 0.025, 0.010 and 0.005 mL were transferred for volumetric flask of 10 mL and solubilised with methanol to obtain concentration of 15.0, 10.0, 5.0, 2.5, 1.0 and 0.5 μg/mL and they were used for the calibration curve. The values of the total phenolic compounds were expressed as gallic acid equivalents (mg of gallic acid (GA)/g of sample) [33].

### LC-Orbitrap-FTMS red propolis EEP

Markers of red propolis quality were identified using high performance liquid chromatography (HPLC) coupled to an ultraviolet detector (Shimadzu). The propolis tincture was prepared at 100 mg/mL in ethanol and diluted to a concentration of 1 mg/mL and used in LC-Orbitrap-FTMS.

The LC-orbitrap-FTMS from Thermo Scientific was used with the following conditions. The stationary phase was a C18 column from ACE (100 × 4.6 mm; 5 μm), and the flow rate was 0.30 mL/min. The mobile phase consisted of (A) 0.1 % formic acid in water: 0.1 % of formic acid in acetonitrile (B) (v:v) in gradient mode. The column was eluted in gradient mode as follows: starting with 30 % of (B), increasing to 45 % in 6 min, 60 in 10 min, 75 in 14 min, 90 in 18 min and 100 in 22 min and held at 100 % B between 22–47 min; then, the gradient decreased to 30 of (B) in 52 min and held at 30 % B between 52–58 min. The FTMS was set to acquire ions in negative mode with a needle voltage of 4.0 kV and sheath gas and auxiliary gas flows of 50 and 10 arbitrary units. The instrument was scanned over the range of 50 to 1200 amu. A volume of 10 μL was injected into the LC-orbitrap-FTMS. The spectra were acquired in negative ion mode with the same source settings as described above and with collision energy of 35 V. Xcalibur 2.2 software from Thermo Fisher Scientific was used to check the raw LC–Orbitrap-FTMS data and generate the MS based chromatograms and masses and formula of the major chromatographic peaks. Xcalibur features from each chromatographic peak was selected based on the peak area and putatively identified by searching for the accurate mass in Dictionary of Natural Products (version 2013), then identified by online library connected to pubchem database.

### Assessment of antioxidant activity by means of the DPPH methods

Quantitative assessment of the antioxidant activity of red propolis EEP and its fractions were performed according to the methods described in the literature [33, 34] with a few modifications. The solvent ethanol was used as blank. The inhibition of free radical DPPH by the samples was monitored by measuring the decrease in absorbance of solutions with different concentrations.

The EEP, hexane, chloroform and ethyl acetate fractions of EEP at an initial concentration of 1.0 mg•mL^−1^ were diluted with ethanol until achieving final concentrations of 25.0, 15.0, 10.0, 5.0 and 2.5 μg•mL^−1^. Then, 1.0 mL of 0.3 mM DPPH in ethanol was added to 2.5 mL of the EEP and it fractions, and the reaction was left to develop in dark at room temperature (26 °C) over 30 min. The absorbance readings were then performed with a spectrophotometer (Model UV-1700, Shimadzu, Kyoto, Japan) at 518 nm.

### Statistical analyses

The mean ± standard deviation of the mean (SDM) relative to the percentage of cell growth inhibition was used to estimate the IC_50_ and determined by means of non-linear regression using software GraphPad Prism, version 5.0. One-factor analysis of variance (ANOVA) followed by Tukey’s or Tamhane’s tests (homogeneous or heterogeneous variance, respectively, Shapiro-Wilk *p* < 0.05) were employed for analysis of the MTT assay results using software Statistical Package for Social Sciences (SPSS, version 21). The significance level was set as *p* < 0.05.

## Results

### Cytotoxicity assay

The first tests, conducted with EEP at a concentration of 50 μg/mL, revealed intense cytotoxic activity against SF-295 (100), OVCAR-8 (93.54) and HCT-116 (98.12 %) cells (Table 1). The cytotoxic effect of EEP did not exhibit relevant variation among the tested cell lines.

**Table 1:** In vitro assessment of Brazilian red propolis cytotoxicity

IC_50_ (μg/mL)			
	Cells		
	HCT-116	SF-295	OVCAR-8
EEP	~25.26	34.27 (24.28 – 48. 37)	28.76 (24.42 – 33.87)
Doxorubicin	0.12 (0.09 – 0.17)	0.24 (0.2 – 0.27)	0.26 (0.17 – 0.30)

Assessment of both Brazilian red propolis extract (EEP) and Doxorubicin cytotoxicity (MTT assay) 72 h after incubation with three tumour cell lines. IC_50_ and 95 % confidence interval - CI (μg/mL) calculated based on the mean and corresponding standard deviation of the mean and determined through non-linear regression

**Fig. 1:**
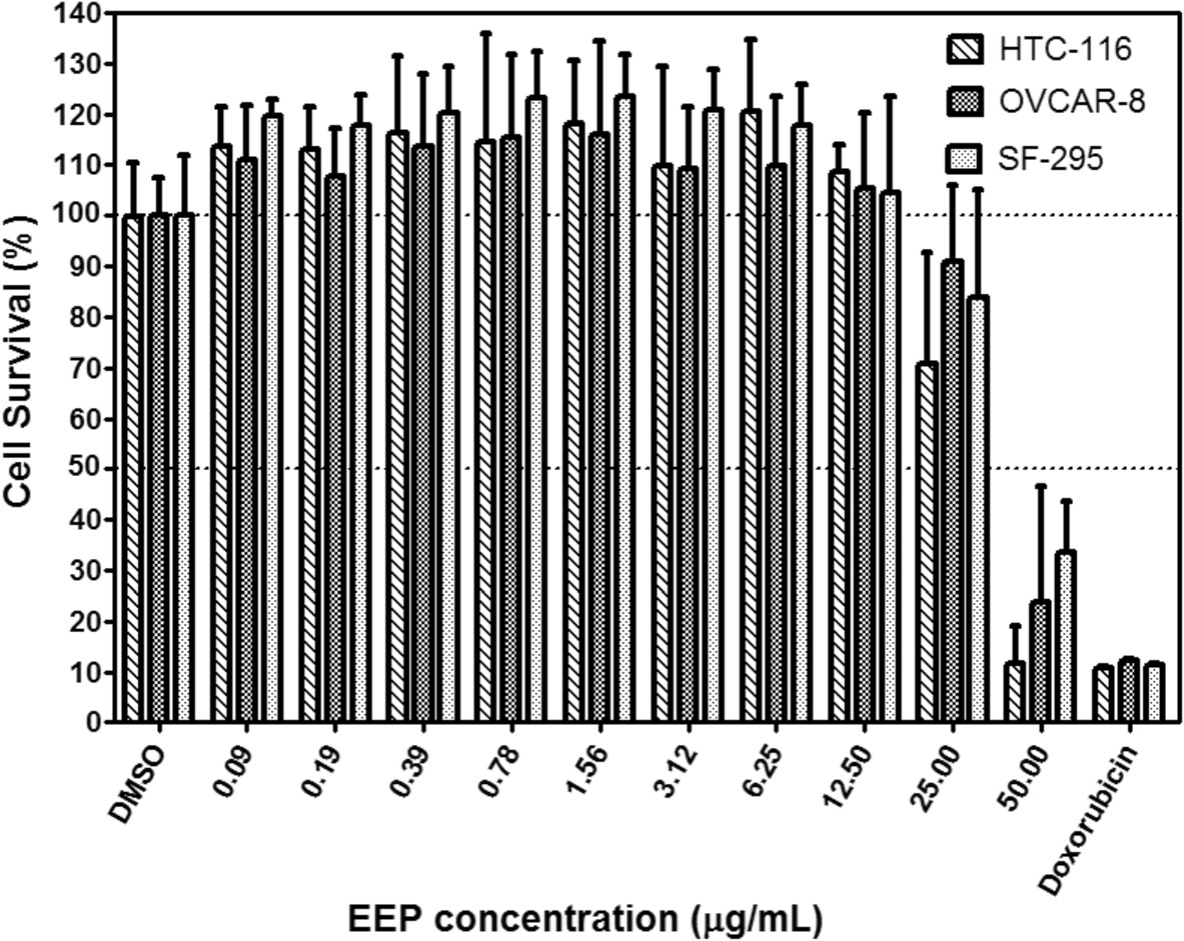
Cell viability 72 h after exposure to various EEP concentrations. Cell survival is expressed as a percentage relative to the negative control (DMSO). EEP at a concentration of 50 μg/mL, exhibited cytotoxic activity against HCT-116 and OVCAR-8 cells, without a significant difference compared with the positive control (0.5 μM doxorubicin). Compared with the other two cell lines, SF-295 cells exposed to EEP at a concentration of 50 μg/mL exhibited a significantly higher survival rate compared with those exposed to doxorubicin. Each bar graph represents the mean, and the error bars represent ± SD of three independent experiments performed in triplicate

Cell viability 72 h after exposure to various EEP concentrations is presented in Fig. 1. From concentrations of 0.09 to 12.5 μg/mL, EEP promoted discrete cell proliferation, which was not significantly different from the negative control. The percentage of viable cells (MTT assay) following exposure to EEP in concentrations up to 25 μg/mL was not significantly different from the negative control. At a concentration of 50 μg/mL, EEP exerted significant cytotoxic activity against all three investigated cell lines, compared to negative control. The viability of HCT-116 and OVCAR-8 cells exposed to EEP (50 μg/mL) did not exhibit significant differences compared with those exposed to doxorubicin, which is a standard anticancer drug. The SF-295 cells exhibited a greater resistance to EEP (50 μg/mL), showing a significant difference compared with the positive control. All three cell-lines exhibited a similar response to the various EEP concentrations, without significant differences among them.

### Phytochemical screening, total phenolic compounds and antioxidant activity of red propolis

**Fig. 2:**
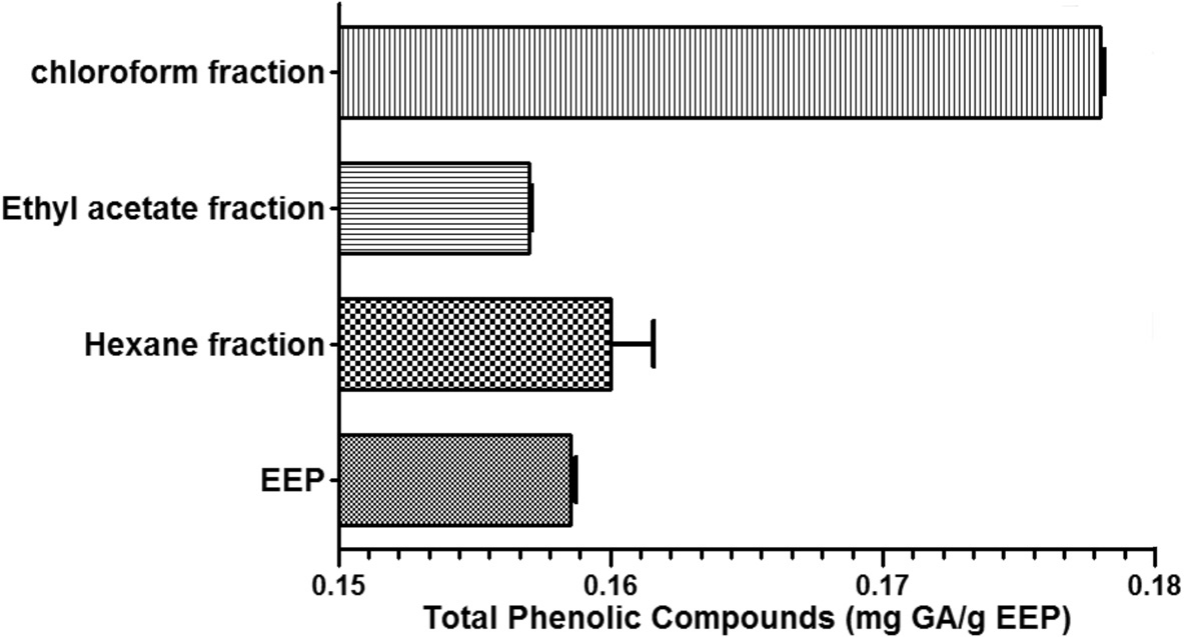
Concentration of phenolic compounds in mg of gallic acid equivalents/g of extract detected in EEP and fractions diluted in hexane, chloroform and ethyl acetate. The highest values corresponded to the chloroform fraction

The presence or absence of the investigated allelochemicals in EEP and its fractions are shown in Table 2. Prospection of the phytochemical components of EEP and its fractions detected the presence of phenolic compounds including flavonoids (catechins, chalcones, aurones, flavones and flavonols), phlobaphene tannins, xanthones and pentacyclic triterpenoids (Table 2). The concentration of the total phenolic compounds in EEP and its fractions is depicted in Fig. 2. The chloroform fraction (0.178 mg GA/g) showed the highest values of total phenolic compounds following by hexane fraction (0.160 mg GA/g) and ethyl acetate fraction (0157 mg GA/g) and EEP (0.1585 mg GA/g).

**Table 2:** Phytochemical screening of the red propolis extract

Allelochemical	EEP	Chloroform fraction	Ethyl acetate fraction	Hexane fraction
Phenols	N^a^	N	N	N
Pyrogallic tannins	N	N	N	N
Phlobaphene tannins	P^a^	P	P	P
Anthocyanin and anthocyanidin	N	N	N	N
Flavones, flavonols and xanthones	P	P	N	P
Chalcones and aurones	P	P	P	N
Flavononols	N	N	N	N
Leucoanthocyanidins	N	N	N	N
Catechins	P	P	P	N
Flavonones	N	N	N	N
Steroids	N	N	N	N
Triterpenoids	P	P	P	P
Saponins	N	N	N	N

Phytochemical screening detected presence of phlobaphene tannins, flavones, flavonols, xanthones and pentacyclic triterpenoids in EEP and its chloroform, hexane and ethyl acetate fractions

^a^ *P* positive, *N* negative

The percentage of antioxidant activity (%AOA) of EEP and the hexane, chloroform and ethyl acetate fractions were assessed by the DPPH method and is shown in Table 3. The antioxidant activity of EEP, ethyl acetate fraction, chloroform fraction and hexane fraction presented IC50 in concentrations 1.8, 2.4, 2.8, 2.8 times, respectively, lower compared to the positive control (trolox) indicating a superior antioxidant activity in relation to trolox drug.

**Table 3:** Antioxidant activity (%AOA) of EEP and its fractions

	Concentration (μg/mL) / (%AOA)						
	25.0	15.0	10.0	5.0	2.5	1.0	IC_50_ (μg/ml)
EEP	86.57	73.43	64.18	38.51	25.97	11.34	8.01
hexane fraction	90.42	87.04	76.62	49.00	44.22	18.87	5.15
chloroform fraction	86.86	84.18	69.55	47.16	41.19	16.79	5.20
ethyl acetate fraction	88.73	76.90	59.44	34.93	24.79	11.27	6.01
Trolox	68.30	50.02	28.00	12.98	6.07	3.02	14.68

Free radical DPPH sequestering activity (%) of the ethanolic extract of Brazilian red propolis and its hexane, chloroform and ethyl acetate fractions

### LC - Orbitrap-FTMS of the red propolis EEP

**Fig. 3:**
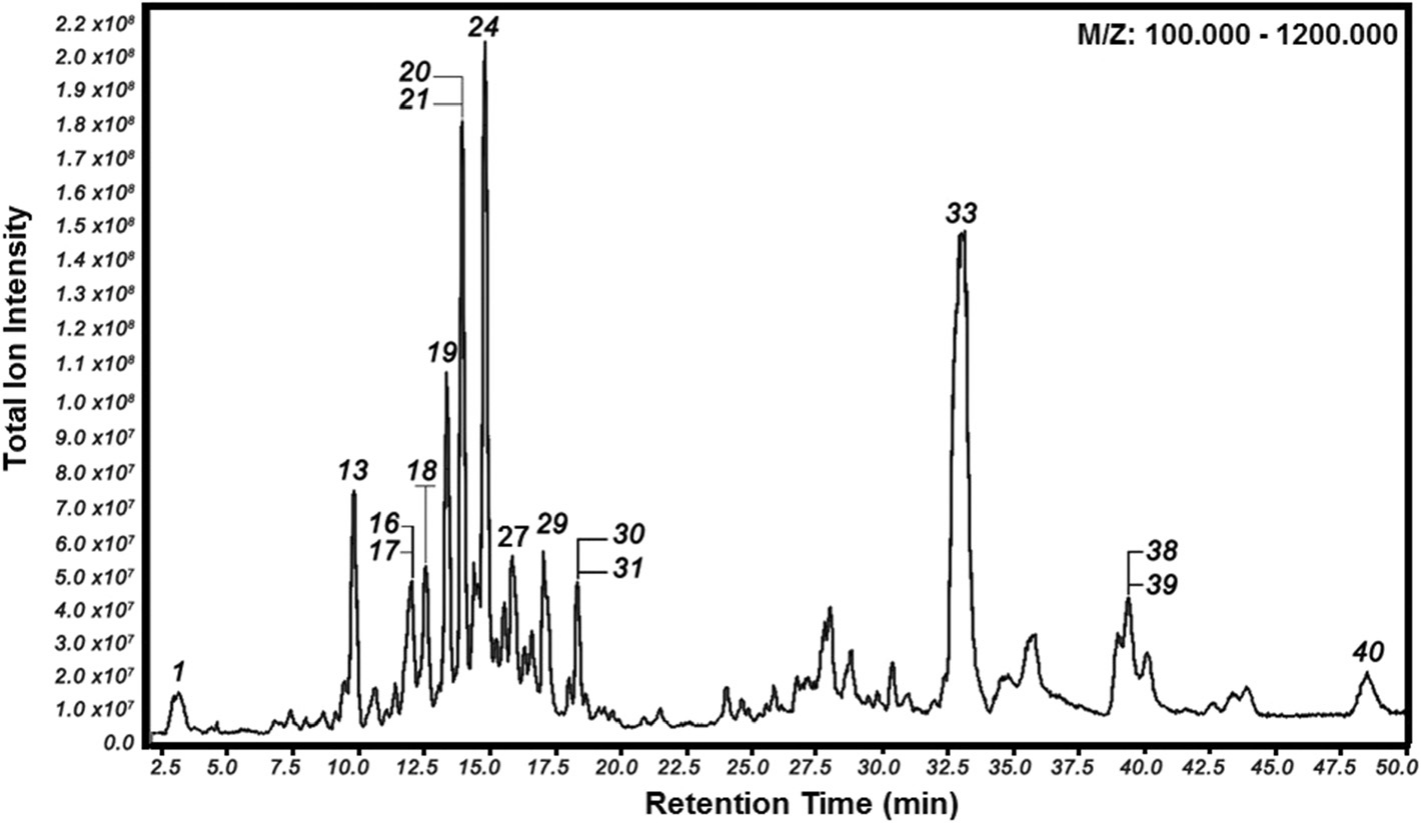
Chromatogram of EEP obtained by means of LC-Orbitrap-FTMS

LC-Orbitrap-FTMS analysis revealed a group of substances that eluted at 3 to 25 min, which corresponds to the group of flavonoids flavonoids such as: phenolic acids, flavan-3-ol (catechins), flavonols, chalcones, isoflavones, isoflavans, pterocarpans and biflavonoids present in Brazilian red propolis, and a group of substances that eluted at 25 to 48 min, which exhibited the characteristic molecular weight of terpens, and guttiferones. The LC-Orbitrap-FTMS chromatogram from EEP is shown in Fig. 3. The main phenolic compounds found in EEP were characterized by LC-FTMS analyses (Table 4). Phytochemical screening (chemical reaction assay) also detected chalcones, flavan-3-ol (catechins), flavonols, but failed to detect other compounds present in EEP such as guttiferones. LC-Orbitrap -FTMS analysis revealed the presence of phlobaphene tannins, guttiferones and some terpenic substances described in Table 3 within the range of 3 to 50 min.

**Table 4:** Identification and confirmation of biomarkers of Brazilian red propolis in EEP using LC-Orbitrap-FTMS

Peak	m/z	RT (min)	Formulae	Compound
1	179.0556	2.95	C_9_H_8_O_4_	Caffeic acid
2	193.0502	2.98	C_10_H_10_O_4_	Ferulic acid
3	179.0556	3.00	C_9_H_8_O_4_	Umbelic acid
4	163.0243	3.04	C_9_H_8_O_3_	p-Coumaric acid
5	475.1232	3.10	C_23_H_24_O_11_	7-O-beta-glucopyranosyl-4′-hydroxy-5-methoxyisoflavone
6	461.1073	4.50	C_22_H_22_O_11_	6-Methoxyluteolin 7-rhamnoside
7	269.0811	7.05	C_15_H_10_O_5_	Genistein
8	285.0395	7.35	C_15_H_10_O_5_	Kaempferol
9	289.0711	8.04	C_15_H_14_O_6_	Cathechin
10	287.0553	8.28	C_15_H_12_O_6_	Dalbergioidin
11	289.0711	8.83	C_15_H_14_O_6_	Epicatechin
12	253.0499	8.95	C_15_H_10_O_4_	Daidzein
13	255.0655	9.7	C_15_H_12_O_4_	Liquiritigenin
14	283.0603	10.5	C_16_H_12_O_5_	2′-Hydroxyformononetin
15	331.0810	11.3	C_17_H_16_O_7_	Evernic acid
16-17	271.0605	11.9	C_15_H_12_O_5_	Naringenin
				Pinobanksin
18	283.0604	12.4	C_16_H_12_O_5_	Calycosin
19	255.0656	13.4	C_15_H_12_O_4_	Isoliquiritigenin
20-21	267.0656	13.8	C_16_H_12_O_4_	Formononetin
				Isoformononetin
22	269.0812	14.2	C_16_H_14_O_4_	4,4′-dihydroxy-2-methoxychalcone
23	269.0812	14.2	C_16_H_14_O_4_	(7S)-dalbergiphenol
24	271.0969	14.7	C_16_H_16_O_4_	Vestitol
25	269.0813	15.1	C_16_H_14_O_4_	Pinostrobin
26	269.0813	15.1	C_16_H_14_O_4_	Medicarpin
27	255.0657	16.2	C_15_H_12_O_4_	2′,6′-dihydroxy-4′-methoxydihydrochalcone
28	255.0657	16.2	C_15_H_12_O_4_	Thevetiaflavone
29	539.1699	17.0	C_32_H_28_O_8_	3′,4′-di-O-benzyl-7-O-(2-hydroxyethyl)-3-O-methylquercetin
30-31	285.1131	18.2	C_17_H_18_O_4_	(3S)-7-O-methylvestitol
				7,3′-Dihydroxy-4′-methoxy-8-methylflavane
32-34	425.1603	21.4	C_30_H_50_O	Cycloartenol
				α-amyrin
				β-amyrin
35-37	601.3533	32.9	C_38_H_50_O_6_	Guttiferone E
				Guttiferone F
				Xantochymol
38-39	669.4156	39.2	C_43_H_58_O_6_	Guttiferone C
				Guttiferone D
40	401.3058	48.4	C_26_H_42_O_3_	19-nor-10-keto-25-hydroxyvitamin D3

## Discussion

The propolis produced in tropical areas contains a wide variety of phenolic compounds, such as p-coumaric acid, flavan-3-ol, flavonols, chalcones, isoflavonoids, pterocarpans, methoxylated isoflavones, triterpenoids and guttiferones [35]. The flavonoids are the main substances that account for the antioxidant [36], anticancer [29, 34, 37] and anti-inflammatory [37] properties of propolis, as well as its protective effects on the kidney, liver and cardiovascular system [36]. Phenolic compounds including formononetin, biochanin A, isoliquiritigenin, pinocembrin and quercetin, the latter in reduced amounts, have been detected in Brazilian red propolis [17]; these compounds exhibit cytotoxic activity against HepG2 (human hepatic carcinoma cells), Hep-2 (human laryngeal epidermoid carcinoma cells) and HeLa (human cervical adenocarcinoma) cancer cell lines [30]. In the present study, the tests to detect phenolic compounds in EEP showed the presence of flavones and flavonols in its chloroform fraction, and the high levels of cytotoxicity exhibited by EEP at a concentration of 50 μg/mL might be attributed to these compounds.

In concentrations up to 12.5 μg/mL, the percentage of viable cells was slightly higher than the negative control. This result might be due to the cytoprotective effects associated with the antioxidant properties of propolis [38]. The cytotoxic activity exhibited a gradual concentration-dependent increase starting at a concentration of 25 μg/mL. The number of surviving cells (HCT-116 and OVCAR-8) in the group exposed to EEP at a concentration of 50 μg/mL was similar to those treated with doxorubicin, a drug routinely used for anticancer treatment. These findings are in agreement with the results of the study conducted by Li et al. [3] using propolis from the same area as in the present study on six different tumour cell lines. In that study, the activity of propolis was similar to that of the investigated anticancer drugs (5-fluorouracil and doxorubicin). An increasing number of epidemiological studies have suggested that flavonoid intake might be associated with a reduced risk of cancer [37]. This observation has been confirmed in *in vitro* [39], *in vivo* [40] and in clinical studies conducted on humans [41].

According to the previous study, propolis interferes with cell replication by inducing apoptosis in cells exhibiting disorganised patterns of growth [42] and also acts in the repair of the damage caused by oxygen free radicals, which might be attributable to the antioxidant activity of its phenolic compounds [30].

Others studies indicated that red propolis induces apoptosis on human tumour cells [2, 12] that might be the mechanism behind the high index of cell inhibition induced by EEP at a concentration of 50 μg/mL showed in this study. Similar effect was also demonstrated by Begnini et al. [22] that found the ethanolic extract of red propolis at concentrations of 50 and 100 μg/mL induced high levels of apoptosis in bladder carcinoma cells.

The cell death type was not assessed in the present study; however, according to Kamya et al. [43], who also tested Brazilian red propolis on tumour cells, EEP significantly reduced the viability of MCF-7 human breast cancer cells through apoptosis triggered by induction of mitochondrial dysfunction, caspase-3 activity and DNA fragmentation.

There is consensus among researchers that reactive oxygen species (reactive molecules and free radicals derived from molecular oxygen) plays a relevant role in mutagenesis and carcinogenesis, based on its ability to damage DNA. Antioxidants play a crucial role in the interception of free radicals and the repair of free radical-induced damage. In addition, that process is related to the removal of damage from the DNA molecule and the reconstitution of damaged cell membranes [44, 45].

In this study the antioxidant activity of EEP and its fractions was determined by *in*-*vitro* antioxidant assay using 2,2-diphenyl-2-picryl-hydrazyl free radical (DPPH) scavenging [46]. The antioxidant action of propolis is attributed to its component flavonoids, among which we might mention quercetin, daidzein, apigenin and genistein [42]. Quercetin and daidzein have been detected by Franchi Jr. et al. [2] in Brazilian red propolis from Alagoas State. Others studies also detected antioxidant activity of the flavonoids isoliquiritigenin [47] and pinobanksin [48] in red propolis.

In the present study, quantitative *in vitro* analysis found that the antioxidant activity of EEP and its fractions (hexane, chloroform and ethyl acetate) were satisfactory, particularly in the case of the chloroform and hexane fractions, being in decreasing order, chloroform fraction > hexane fraction > EEP ≅ ethyl acetate fraction. The greater antioxidant activity of the chloroform and hexane fractions correlates with the higher concentration of phenolic compounds in those fractions compared with the other. These results are in agreement with those reported by Cabral et al. [46] upon assessing the antioxidant activity of Brazilian red propolis.

Chloroform fraction and hexane fraction were enriched with phenolic compounds of red propolis during liquid-liquid extraction and was dependent of the partition coefficient (K) of the phenolic compounds in the solvents chosen for this procedure of extraction. The hexane fraction presented the second highest total phenolic compound values. This can be justified due to partition coefficient (K) of these compounds resulting in the migration of phenolic compounds during partitioning process of liquid-liquid extraction [49]. Plus the lack of selectively of the hexane and chloroform solvents for phenolic compounds present in red propolis, which present an semi-polar nature such as methoxylated isoflavonoids (formononetin) and prenylated phenolic compound (guttiferone) (Table 4) and xanthones (Table 2) besides of phlobaphene tannins (Table 2) in all samples detected in phytochemical prospection.

Generally, phytochemicals prospection assays are qualitative, quick and low-cost methods and assure presence or absence of different classes of secondary metabolites. But an aspect to be considered is that the crude extract (EEP) can present various substances of polar, semi-polar, and non-polar nature with much interference and become the sample very complex to detect presence of compounds. Other aspect to be considered is the lack of selectivity of the solvents and can be a limiting factor on the assay. This lack of selectivity of the solvents also can be explained by the theory of partition coefficient (K) of the analytes. Analytes can be partitioned by solvents and depending on: 1) concentration of analytes; 2) solubility of analytes in solvent of extraction; 3) volume of the solvent of extraction; 4) the nature of analytes (acid or base); 5) pH of the aqueous phase.

In our experiment, the extraction of phenolic compounds by hexane solvent was conducted by 1) Large amount of the crude mass (crude extract) resulting in low concentration of crude mass after first liquid-liquid partitioning (theorical concentration of crude mass was less than 100 mg/mL); 2) and volume of 100 mL (large amount of solvent of extraction, hexane solvent); 3) no control of pH in aqueous medium and it favored the partitioning process of liquid-liquid extraction in non-polar solvent (hexane) despite phenolic compound have a limited solubility by hexane solvent.

The modern technique of phytochemical prospection using LC-Orbitrap-FTMS demonstrated to be more specific in the identification of the exact biomarkers of red propolis independently of the class of secondary metabolites (biomarkares). It was possible to detect the presence of polyisoprenylated guttiferones (35–39), terpens (32–34), pterocarpan (26), isoflavans (24), isoflavones (20–21), chalcone (19), flavonone (16), dihydroflavonol (17), flavonols (8), phenolic acids (9), flavans (4) (See Table 4) and which are considered as bioactive markers in this type of Brazilian propolis. Tannins and xanthones are phenolic compounds with remarkable antioxidant activity [50]. The presence of tannins and xanthones along with the flavonoids might also account for the antioxidant and anticancer activity exhibited by EEP in this study [30, 38, 42, 46].

LC-Orbitrap-FTMS can be considered the best choice to detect any substance because MS is a universal detector in large range of masses. LC-Orbitrap-FTMS is more sensitive and robust for detecting different compounds in different range of masses because Orbitrap is an important component in this equipment because can concentrate the ions during the detection. For LC-Orbitrap-FTMS a concentration of 1 mg/mL (1000 μg/mL) of crude extract was prepared and only 10uL (10 μg) was injected in the chromatographic column. This amount is enough to detect in the mass spectrometer using the orbitrap mode due to the sensibility of the technique to detect a hundred compounds in simple analysis [51]. Thus it is the best choice technique to evaluate the chemical profile analysis and modern fingerprint of complex samples like phytochemicals and apiceuticals.

## Conclusions

It was possible to confirm the presence of phlobaphene tannins, catechins, flavones, flavonols, chalcones, isoflavans, pterocarpans, isoflavonoids, biflavonoids, pentacyclic triterpenoids, xanthones and guttiferones in EEP using the classical chemical prospection and a modern analytical technique such as LC-Orbitrap-FTMS. LC-Orbitrap-FTMS was useful to establish the chemical profile of the red propolis. Brazilian red propolis exhibited antioxidant activity and in higher concentration it showed high cytotoxic potential on the human tumour cell lines SF-295, HCT-116 and OVCAR-8. Therefore, it might be useful for the development of new medicines and phytomedicines for the treatment of cancer.

## Abbreviations

SF-295: Tumour cell line of human glioblastoma (from National Cancer Institute-USA)
OVCAR-8: Tumour cell line of ovary (from National Cancer Institute-USA)
HCT-116: Tumour cell line of colon (from National Cancer Institute-USA)
EEP: Ethanolic extract of Brazilian red propolis
DPPH: 2,2-diphenyl-1-picryhydrazyl
MTT: Salt 3-(4,5-dimethyl-2-thiazole)-2,5-diphenyl-2-H-tetrazolium bromide
RPMI 1640: Roswell Park Memorial Institute medium
FBS: Foetal bovine serum
DMSO: Dimethyl sulphoxide
%AOA: Percentage of antioxidant activity
(K): Partition coefficient
LC-orbitrap-FTMS: High performance liquid chromatography with technology Orbitrap and Fourier Transform Mass Spectrometer
